# Discovery of Novel Inhibitors and Fluorescent Probe Targeting NAMPT

**DOI:** 10.1038/srep12657

**Published:** 2015-07-31

**Authors:** Xia Wang, Tian-Ying Xu, Xin-Zhu Liu, Sai-Long Zhang, Pei Wang, Zhi-Yong Li, Yun-Feng Guan, Shu-Na Wang, Guo-Qiang Dong, Shu Zhuo, Ying-Ying Le, Chun-Quan Sheng, Chao-Yu Miao

**Affiliations:** 1 Department of Pharmacology, Second Military Medical University, Shanghai, China; 2 Department of Medicinal Chemistry, Second Military Medical University, Shanghai, China; 3 Key Laboratory of Food Safety Research, Institute for Nutritional Sciences, Shanghai Institutes for Biological Sciences, Chinese Academy of Sciences.; 4 Key Laboratory of Food Safety Risk Assessment, Ministry of Health, Beijing 100021, China

## Abstract

Nicotinamide phosphoribosyltransferase (NAMPT) is a promising antitumor target. Novel NAMPT inhibitors with diverse chemotypes are highly desirable for development of antitumor agents. Using high throughput screening system targeting NAMPT on a chemical library of 30000 small-molecules, we found a non-fluorescent compound F671-0003 and a fluorescent compound M049-0244 with excellent *in vitro* activity (IC_50_: 85 nM and 170 nM respectively) and anti-proliferative activity against HepG2 cells. These two compounds significantly depleted cellular NAD levels. Exogenous NMN rescued their anti-proliferative activity against HepG2 cells. Structure-activity relationship study proposed a binding mode for NAMPT inhibitor F671-0003 and highlighted the importance of hydrogen bonding, hydrophobic and π-π interactions in inhibitor binding. Imaging study provided the evidence that fluorescent compound M049-0244 (3 μM) significantly stained living HepG2 cells. Cellular fluorescence was further verified to be NAMPT dependent by using RNA interference and NAMPT over expression transgenic mice. Our findings provide novel antitumor lead compounds and a “first-in-class” fluorescent probe for imaging NAMPT.

Nicotinamide phosphoribosyltransferase (NAMPT) plays a key role in mammalian nicotinamide adenine dinucleotide (NAD) biosynthesis^1^. NAMPT catalyzes the conversion of nicotinamide into nicotinamide mononucleotide (NMN), which is subsequently transformed to NAD under the catalysis of nicotinamide mononucleotide adenylyltransferase (NMNAT). Due to its importance in cellular physiopathological process and close relationship with occurrence and development of tumor, it becomes a promising target for the discovery of novel antitumor agents^2^,^3^,^4^,^5^.

Up to now, several classes of NAMPT inhibitors have been reported^6^. Among them, CHS-828^7^ and FK866^8^,^9^, two most advanced compounds, have been progressed to clinical trials. However, their clinical outcome is disappointing. No significant patient responses were observed for both of them^9^. Furthermore, CHS-828 and FK866 suffered from unfavorable pharmacokinetic properties (*e.g.* low bioavailability, rapid intravenous clearance, and large data variation) and severe dose-limiting side effects (*e.g.* trombocytopoenia and gastrointestinal toxicity)^8^,^9^. To overcome the drawbacks of CHS-828 and FK866, a number of structural analogues have been reported^6^,^10^. Notably, several of them showed improved pharmacokinetic profiles and significant *in vivo* antitumor potency in human glioblastoma, ovarian carcinoma, fibrosarcoma xenograft models^11^,^12^,^13^. These data supported the fact that NAMPT inhibitors might have an important therapeutic role in the treatment of cancer. Even though, much work remains to be done in this area. Considering limited structural classes of the reported NAMPT inhibitors, the discovery of diverse inhibitors with novel chemotypes is still highly desirable to validate the druggability of NAMPT as an antitumor target. Moreover, the identification of novel chemical tools, particularly fluorescent probes, is also important to the better understanding of the biological function of NAMPT.

Herein, structurally diverse NAMPT inhibitors were identified by high throughput screening. In particular, we developed a first-in-class fluorescent probe to straight detection and snapshot of NAMPT at molecular and cellular level, which may provide a novel and convenient strategy to study NAMPT.

## Results

**Figure 1:**
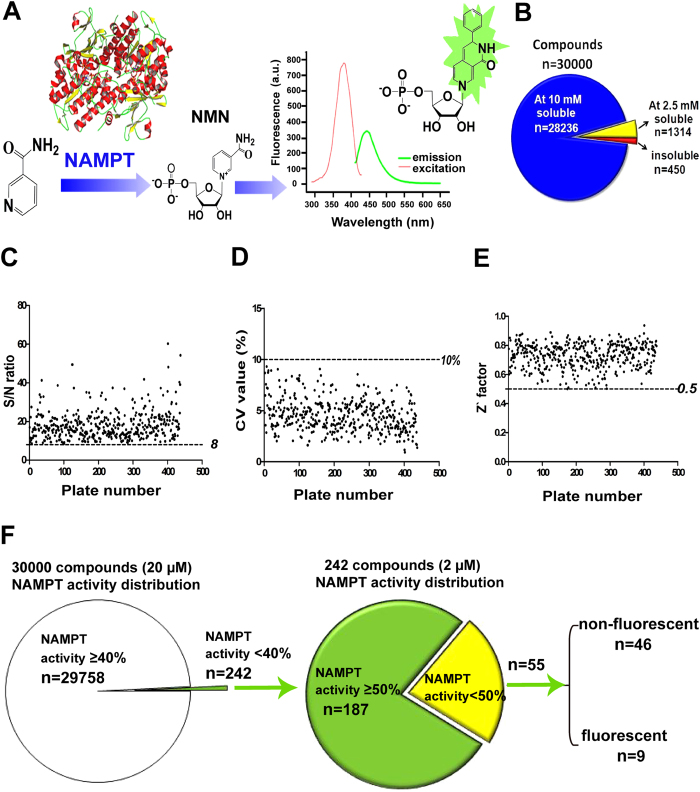
Discovery of structurally diverse NAMPT inhibitors by HTS in a chemical library containing 30000 small-molecule compounds. (**A**) Schematic illustration of detecting NAMPT activity. (**B**) Solubility of 30000 compounds. (**C**–**E**) The signal-to-noise (S/N) ratio, coefficient of variation (CV) value and Z’ factor used for evaluation of HTS assays. (**F**) Schematic illustration of discovering structurally diverse NAMPT inhibitors by HTS.

Using our previously established high throughput screening (HTS) platform and recombinant human NAMPT^14^,^15^, we performed a HTS on a chemical library containing 30000 small-molecules (Chemdiv, CA, USA). Our HTS targeting NAMPT was developed based on a fluorometric method for NAMPT activity assay by measuring the fluorescence of nicotinamide mononucleotide (NMN) derivative resulting from the NAMPT enzymatic product NMN through simple chemical reactions (Fig. 1A). The 30000 compounds were dissolved with dimethyl sulfoxide (DMSO); most of compounds were soluble at 10 mM stock solution (94.1%), some compounds insoluble at 10 mM were soluble at 2.5 mM stock solution (4.4%), and the remaining compounds insoluble at 2.5 mM (1.5%) were diluted further during HTS experiments (Fig. 1B). To guarantee the quality of screening, S/N ratio, CV and Z’ factors were monitored throughout the screenings, and all three indices met the requirements of HTS (Fig. 1C–E). After a primary screen and a secondary screen of all compounds at 20 μM, 242 compounds were selected as the hits of NAMPT inhibitors (NAMPT activity < 40%), and the hit rate was ~0.8% (Fig. 1F). Further, using three concentrations (2, 0.2 and 0.02 μM) to examine the effect of 242 compounds on NAMPT activity, we found 55 compounds reducing NAMPT activity to less than 50% at 2 μM, suggesting these compounds have a potent NAMPT inhibition. All these 55 compounds were not reported as NAMPT inhibitors previously. Among the 55 compounds, we noted 46 compounds were non-fluorescent and 9 compounds were fluorescent (Fig. 1F). These fluorescent and non-fluorescent compounds were further studied separately.

**Figure 2:**
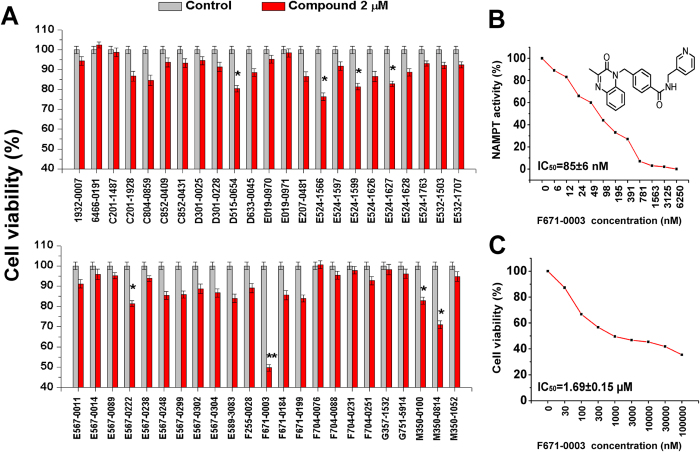
Effects of 46 non-fluorescent compounds on NAMPT activity and cell viability. (**A**) Effects of 46 compounds at 2 μM on HepG2 cell viability. (**B**) Concentration response curve of F671-0003 on NAMPT activity. (**C**) Concentration response curve of F671-0003 on HepG2 cell viability. Data are shown as mean ± SEM. ^*^P < 0.05, ^**^P < 0.01 vs. Control.

IC_50_ values for NAMPT inhibition of 46 non-fluorescent compounds were all less than 2 μM, calculated by study of concentration response relationships (Table 1, Supplemental dataset S1). In cell experiments, 8 of 46 NAMPT inhibitors demonstrated a significant inhibition on cell viability (Fig. 2A), after 48 hours of incubation with HepG2 cells, a human hepatocellular carcinoma cell line with abundant NAMPT expression (Figure S1). Among these 8 compounds (D515–0654, E524–1566, E524–1599, E524–1627, E567-0222, F671-0003, M350-0100 and M350–0814), F671-0003 was the most effective NAMPT inhibitor (Fig. 2A). The IC_50_ value of F671-0003 was 85 ± 6 nM for NAMPT inhibition in molecular/biochemical assays (Fig. 2B), and F671-0003 at 2 μM produced a 50% inhibition on cell viability in cellular assays (Fig. 2A). Further, we gave a concentration response relationship in cellular assays (Fig. 2C); the IC_50_ value was 1.69 ± 0.15 μM for the inhibitory effect of this compound on cell viability.

**Figure 3:**
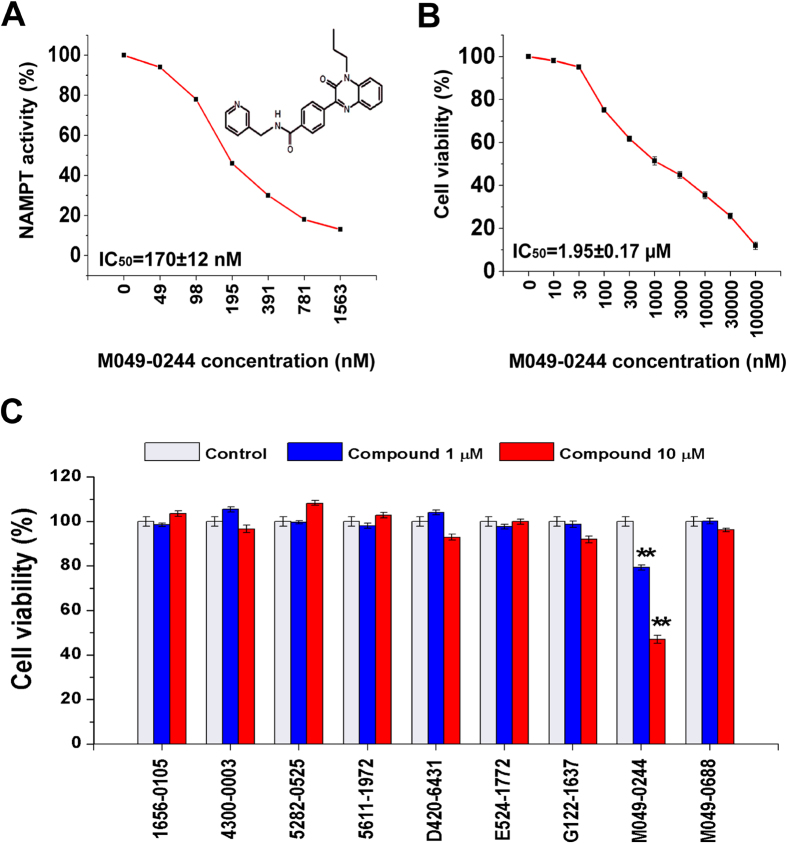
Effects of 9 fluorescent compounds on NAMPT activity and cell viability. (**A**) Concentration response curve of M049-0244 on NAMPT activity. (**B**) Concentration response curve of M049-0244 on HepG2 cell viability. (**C**) Effects of 9 compounds at 1 μM and 10 μM on HepG2 cell viability. Data are shown as mean ± SEM. ^**^P < 0.01 vs. Control.

For 9 fluorescent compounds, concentration response relationships demonstrated that 4 compounds (1656-0105, 4300-0003, 5282-0525 and 5611-1972) were not appropriate for IC_50_ determination using our biochemical assays for NAMPT activity (Supplemental dataset S2). However, IC_50_ values for other 5 fluorescent compounds were able to be determined (Supplemental dataset S2). We then measured the fluorescence of all 9 fluorescent compounds at a series of different concentrations. It was demonstrated that the fluorescence of 4 compounds (1656-0105, 4300-0003, 5282-0525 and 5611-1972) was very strong and detected at very low concentrations (3–24 nM), leading to inappropriate for IC_50_ determination of these 4 chemicals. In contrast, the fluorescence of other 5 compounds (D420-6431, E524-1772, G122-1637, M049-0244 and M049-0688) was relatively weak and detected at higher concentrations (>1562 nM, Supplemental dataset S2). Therefore, we used the concentrations with undetected fluorescence (≤1562 nM) to make concentration response curves for determining IC_50_ of these 5 compounds; the IC_50_ values were 415 ± 208 nM, 33 ± 1 nM, 411 ± 148 nM, 170 ± 12 nM and 365 ± 122 nM respectively (Fig. 3A, Table 1, Supplemental dataset S2). Cell experiments using two concentrations (1 and 10 μM) showed that one compound (M049–0244) produced a significant inhibition on HepG2 cell viability in a dose dependent manner, while other 8 compounds had no effects (Fig. 3C). These data exclude the importance of 8 fluorescent compounds in living cells although they had NAMPT inhibition activity at molecular level. Further, we determined the concentration response relationship of the effective compound (M049-0244) in cellular assays; the IC_50_ value was 1.95 ± 0.17 μM for the inhibitory effect of this compound on HepG2 cell viability (Fig. 3B). Inhibitory effects of M049–0244 and F671-0003 on cell viability were also found on Huh-7 cell, another human hepatocellular carcinoma cell line (Figure S2).

Next, we performed structure-activity relationship (SAR) study. A total of 454 structural analogues of the 46 non-fluorescent NAMPT inhibitors (Supplemental dataset S1, Supplemental dataset S3) were selected within the present chemical library and re-checked for NAMPT inhibition activity. 437 compounds (96.3%) showed no NAMPT inhibition activity at 20 μM (NAMPT activity ≥40%), 12 compounds (2.6%) had lower NAMPT inhibition activity (2 μM < IC_50 _< 20 μM), and 5 compounds (1.1%) including 3 fluorescent compounds and 2 non-fluorescent compounds had NAMPT inhibition activity. These data are consistent with the abovementioned results, further supporting that our HTS is reliable. Our data also provide information for no use of these ineffective chemicals in the development of NAMPT inhibitors (Supplemental dataset S1, Supplemental dataset S3).

**Figure 4:**
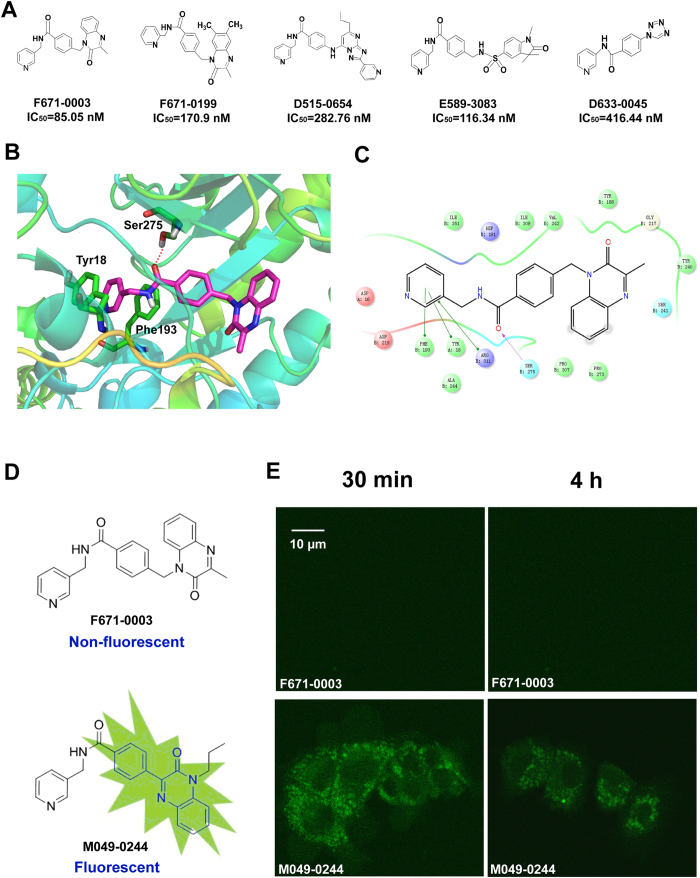
Structure-activity relationship (SAR) and binding mode of NAMPT inhibitors and imaging study of fluorescent molecule M049-0244 in living cells. (**A**) SAR analysis of F671-0003 analogues. (**B**–**C**) Binding mode analysis of F671-0003 with NAMPT. (**D**) Non-fluorescent NAMPT inhibitor (F671-0003) and fluorescent NAMPT inhibitor (M049–0244) are a pair of analogues. (**E**) Live cell fluorescence microscopy of HepG2 cells exposed to the compounds at 3 μM.

More specifically, we performed SAR analysis for NAMPT inhibitor F671-0003, and proposed a binding mode (Fig. 4A–C). Among the novel NAMPT inhibitors identified from HTS, compound F671-0003 showed a very effective inhibitory activity according to the combined molecular and cellular data (Fig. 2, Table 1 and Fig. 4A). To investigate the binding mode of F671-0003, it was docked into the active site of NAMPT. As shown in Fig. 4B,C, compound F671-0003 interacted with an extended conformation. The pyridinyl group formed face-to-face π-π interactions with Phe193 and Tyr18, respectively. The carbonyl oxygen atom formed hydrogen bonding interaction with the side chain of Ser275. The phenyl group attached to the amide interacted with Ile309 and Val242 mainly through hydrophobic and van der Waals interactions. The terminal quinoxalinone ring was located at the outside of active site and faced to the solvent. The SAR of F671-0003 was further investigated by analyzing its structural analogues listed in Fig. 4A. Chemical structure of F671-0199 is very similar to that of F671-0003. However, the movement of pyridinyl nitrogen atom and addition of the two methyl of quinoxalinone group led to obvious decrease of the NAMPT inhibitory activity possibly because the reduced π-π interactions with Phe193 and Tyr18. For compound D515-0654, its triazolopyrimidine group is a mimic of quinoxalinone group in compound F671-0003. However, its propyl substitution might have steric clash with the pocket lined with Gly217, Tyr240 and Ser241. More the pyridinyl substitution attached at the triazoyl ring was exposed to the solvent and had little effect on inhibitor binding. Thus, compound D515-0654 was less active than F671-0003. As compared with F671-0003, compound has longer terminal side chain, which was exposed to the solvent. Due to decreased interaction between the side chain and its surrounding residues (*e.g.* Pro273, Pro307 and Tyr240), E589–3083 showed weaker NAMPT inhibitory activity than F671-0003. Similarly, inhibitor D633-0045 only has a small tetrazole substitution attached to the amide phenyl group, which significantly decreased the interaction with the surrounding residues. As a result, it was the weakest inhibitor among its analogues.

Also, SAR study demonstrated an unexpected fact that a non-fluorescent NAMPT inhibitor (F671-0003) and a fluorescent NAMPT inhibitor (M049–0244) are a pair of analogues (Fig. 4D). The fluorescent molecule M049–0244 has a 3-phenylquinoxalin-2(1*H*)-one group (HNQ), which was reported to be a fluorophore^16^. In contrast, its structural analogue F671-0003 is a non-fluorescent molecule because the methene between the phenyl and quinoxalin-2(1*H*)-one group disrupted the π-conjugated system. Based on the docking model, the fluorescent NAMPT inhibitor M049-0244 might share similar binding mode to F671-0003. Importantly, in cellular assays their potency (IC_50_) was around 2 μM, similar for both compounds, while the efficacy (maximal effect) was better for M049–0244 than F671-0003 (Fig. 2C and Fig. 3B).

As we know, small-molecule fluorescent probes have been widely used to image and detect bio-targets. However, no fluorescent probe for imaging and detecting NAMPT has been reported up to date. Thus, we evaluated whether M049-0244 can be applied for imaging the NAMPT in living cells. Based on time course and dose dependent experiments (Figure S3), we found that the fluorescence was nearly saturated in HepG2 cells after incubated with M049–0244 for 30 minutes. The fluorescence intensity did not show significant increase at 10 μM compared with 3 μM. Therefore, we chose 3 μM concentration and 30 minutes incubation in imaging experiments. The fluorescence was obviously detected within the cell, after incubation with M049-0244 (3 μM) for 30 minutes and wash with fresh culture medium completely (Fig. 4E). And, the fluorescence was stable in the cell with no significant extracellular release after additional culture for several hours (Fig. 4E). Meantime, F671-0003 (3 μM) or DMSO (vehicle) did not show any fluorescence under the same conditions. The results reveal that this fluorescent probe can significantly stain HepG2 cells, which could directly monitor NAMPT temporally and spatially.

**Figure 5:**
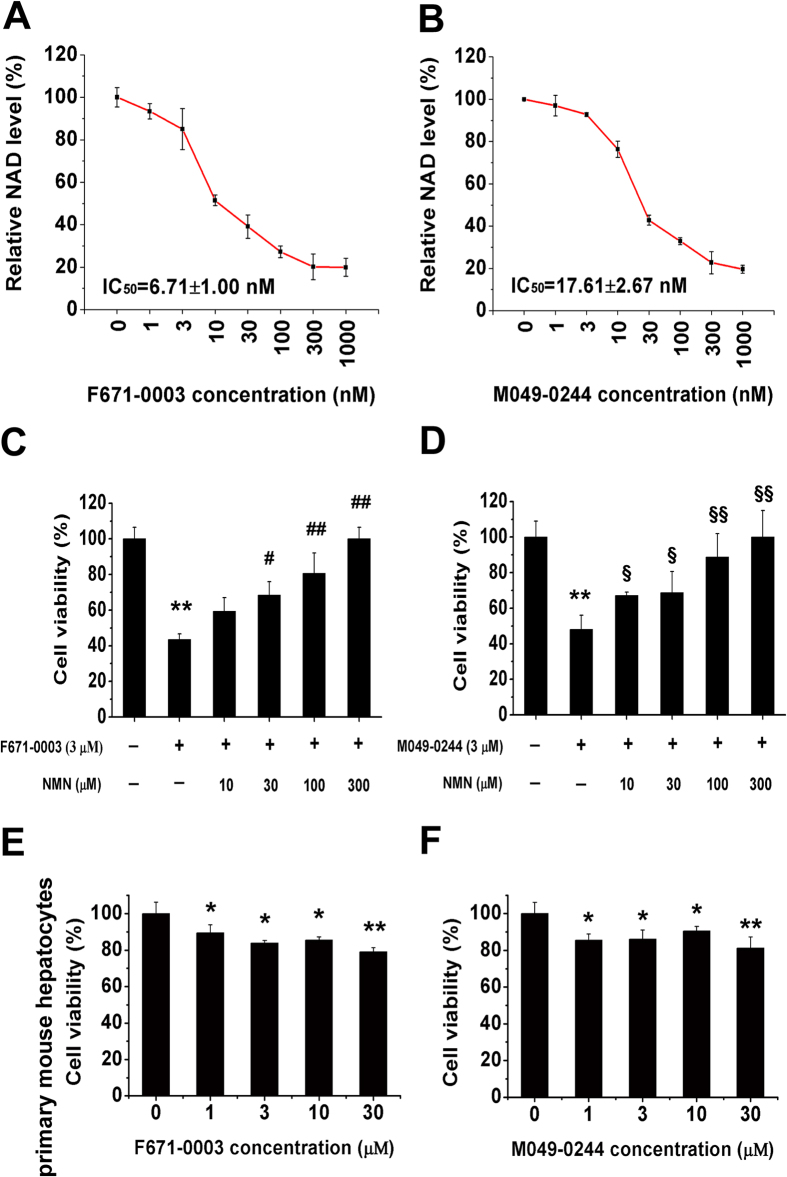
Monitoring target specificity and cellular specificity of F671-0003 and M049-0244. Concentration response curve of F671-0003 (**A**) and M049–0244 (**B**) on HepG2 NAD levels after 24 hours treatment. NMN rescued the inhibition of F671-0003 (**C**) and M049–0244 (**D**) on cell viability. ^*^P < 0.05, ^**^P < 0.01 vs serum free medium control; ^#^*P *< 0.05, ^##^*P *< 0.01 vs 3 μM F671-0003 without NMN. ^§^*P *< 0.05, ^§§^*P *< 0.01 vs 3 μM M049-0244 without NMN. Effect of F671-0003 (**E**) and M049-0244 (**F**) on primary mouse hepatocytes viability. *P < 0.05, **P < 0.01 vs serum free medium control. Data are shown as mean ± SEM.

To validate the target specificity of F671-0003 and M049-0244 in intact cell, we determined NAD levels and performed NMN rescue studies. After incubation with human HepG2 cells for 24 hours, F671-0003 and M049-0244 both decreased the cellular NAD level by ~80% at 1 μM (Fig. 5A,B). The IC_50_ for F671-0003 reducing NAD level was 6.71 nM (Fig. 5A) and for M049-0244 was 17.61 nM (Fig. 5B). Besides, NMN supplement also rescued the cell viability. As shown in Fig. 5C,D, exogenous NMN dose dependently reversed the inhibition of cell viability caused by F671-0003 or M049–0244. NMN at 300 μM totally rescued the inhibition of cell viability caused by F671-0003 or M049–0244. We further observed their inhibitory effect on normal hepatocytes. Results showed that F671-0003 and M049–0244 had mild inhibition on primary hepatocytes viability from normal mice (Fig. 5E,F). They inhibited normal hepatocytes viability by ~20% at 30 μM, much weaker than those on HepG2 cells (Fig. 2C and Fig. 3B).

**Figure 6:**
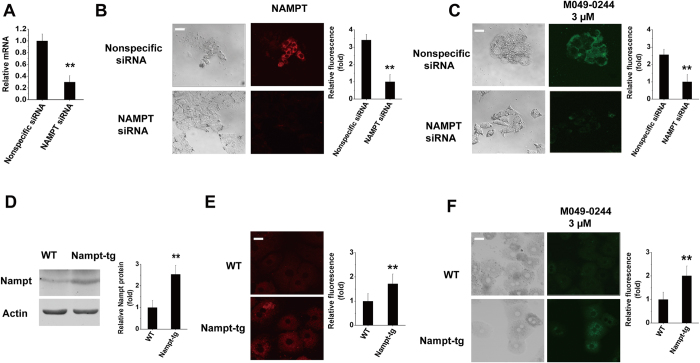
Monitoring fluorescence specificity of M049-0244. (**A**) Knockdown efficiency was confirmed by quantitative PCR. The mRNA levels were normalized to actin. **P < 0.01 vs nonspecific siRNA. (**B**) Immunofluorescent staining of NAMPT in HepG2 cells transfected with nonspecific siRNA or NAMPT-specific siRNA. **P < 0.01 vs nonspecific siRNA. (**C**) Live cell fluorescence microscopy of HepG2 cells transfected with nonspecific siRNA or NAMPT-specific siRNA exposed to M049–0244 (3 μM). **P < 0.01 vs nonspecific siRNA. (**D**) Western-blot analysis of NAMPT expression in liver of wild type and NAMPT over expression-transgenic mice. **P < 0.01 vs wild type. (**E**) Immunofluorescent staining of NAMPT in primary mouse hepatocytes from WT or NAMPT over expression-transgenic mice. **P < 0.01 vs wild type. (**F**) Live cell fluorescence microscopy of primary mouse hepatocytes from wild type or NAMPT over expression-transgenic mice. **P < 0.01 vs wild type. Data are shown as mean ± SEM. Bar = 20 μm.

To demonstrate the fluorescence specificity of M049–0244, we used siRNA to knockdown NAMPT in HepG2 cells and NAMPT over expression transgenic mice^17^ for primary hepatocytes culture. Real-time PCR and immunofluorescent staining showed efficient knockdown of NAMPT (about ~70%) by siRNA targeting NAMPT (Fig. 6A,B). As shown in Fig. 6C, RNAi-NAMPT significantly reduced the fluoresecence induced by M049-0244 at 3 μM in HepG2 cells. Further, western-blot analysis and immunofluorescent staining verified NAMPT overexpression in liver cells of transgenic mice (Fig. 6D,E). We then compared the cellular fluoresecence intensity induced by M049–0244 at 3 μM from primary cultured wild type mice hepatocytes and NAMPT over expression transgenic mice hepatocytes. Apparently, NAMPT over expression increased the cellular fluorescence intensity induced by M049-0244 (Fig. 6F).

## Discussion

Currently, there are two HTS methods to discover NAMPT inhibitors. One is our *in vitro* screening target NAMPT^14^, the other is cellular phenotypic screening^10^. Our HTS method targeting NAMPT is sensitive, simple, quick and cost-effective. It is convenient for analysis of SAR of chemical compounds, which is very important for later optimization^15^. Moreover, this method can be not only applied to discover inhibitors, but also applied to discover activators. Up to now, we have screened ~55,000 compounds in the previous^14^,^15^ and present works. Among them, at the concentration of 20 μM, there are 348 compounds with NAMPT activity <40%, and 495 compounds with NAMPT activity >125%. Further studies will be done to verify the NAMPT activity of 495 potential active compounds *in vitro* and *in vivo*.

One important finding of the present study is discovering novel structurally diverse NAMPT inhibitors. Especially, F671-0003 and M049–0244 have the best cellular activity. According to the previous reports, chemical compounds verified to bind with desired targets *in vitro* do not necessarily bind with the same target in intact cell or *in vivo*. As a case in point, a proposed PARP-1 inhibitor iniparib reached phase III clinical trials, where it showed no efficacy, and was subsequently shown to lack activity against PARP-1 in living cells^18^. Because NAMPT is the rate-limiting enzyme in NAD salvage pathways, we further verified the target specificity by function measurement. F671-0003 and M049–0244 both can significantly down-regulate NAD levels in HepG2 cells. Moreover, supplement with NMN, a direct enzymatic product of NAMPT, can rescue the cell viability in the existence of the two compounds. These results support the two compounds acting on NAD salvage pathway driven by NAMPT in intact cells. There are two enzymes, NAMPT and NMNAT, in the NAD salvage pathway. NAMPT catalyzes the conversion of nicotinamide into nicotinamide mononucleotide (NMN), which is subsequently transformed to NAD by NMNAT. The use of NMN rather than NAD in the rescue studies excluded the possible inhibition of these two compounds on NMNAT.

Considering anti-tumor compounds may have cytotoxity on normal cells, we tested these two compounds in primary normal cells. Our results showed that F671-0003 and M049-0244 had mild inhibition on normal liver cell viability, much weaker than those on hepatocellular carcinoma cells. This is in agreement with previously reported NAMPT inhibitors, such as FK866^19^. Most normal cells are insensitive to NAMPT inhibitors, except those have high NAD cleaving reactions such as lymphocytes^20^. NAD regeneration is primarily mediated through NAMPT. Tumors have elevated poly (ADP-ribose) polymerase activity and thereby consume NAD at a higher rate than normal tissues and are thus more dependent upon NAMPT to maintain required NAD levels^2^. Accordingly, NAMPT inhibitors show stronger cytotoxity on tumor cells compared with normal cells.

Molecular docking studies revealed that the F671-0003 and M049-0244 bound with the same pocket to that of FK866 and CHS828 in the active site of NAMPT (Figure S4). Although the NAMPT inhibitory activity of these novel inhibitors was still lower than FK866, they represent novel chemical scaffolds for further structural optimization to discover highly potent inhibitors. We calculated the similarity index of inhibitors F671-0003 and M049–0244 with the NAMPT inhibitors reported in a recent review^21^ (Table S1). The results indicated that the two compounds shared low structural similarity with the known NAMPT inhibitors. Moreover, the physicochemical properties of inhibitors F671-0003 and F671-0003 were calculated (Table S2). Interestingly, the logP value of inhibitor F671-0003 is lower than CHS-828 and FK866, indicating that it might have better solubility and oral bioavailability. Structural optimization of these inhibitors as well as their pharmacokinetic profiles and *in vivo* antitumor potency remain to be further investigated.

Another important finding in this study is providing a fluorescent probe M049–0244 for imaging NAMPT in living cells. Except for the biological functional evidence, we also provided the fluorescent specificity evidence for M049–0244 using immunofluorescence, RNA interference and NAMPT over expression transgenic mice. Although major fluorescence in cells was dependent on NAMPT, some fluorescence may be independent of NAMPT due to possible nonspecific distribution or binding. NAMPT is dominantly expressed in the cytoplasm. NAMPT immunofluorescence and cellular fluorescence stained by M049–0244 from our studies showed the similar distribution. NAMPT is also found in mitochondrial in many types of cells^22^,^23^. And, FK866, a well known NAMPT inhibitor was reported to distribute in mitochondrial as well as cytoplasm^24^. However, whether M049–0244 distributes to specific organelles such as mitochondrial still needs further investigation.

In summary, novel NAMPT inhibitors were identified by a HTS study. The structural classes and SAR information for the NAMPT inhibitors were significantly expanded, which were important for discovering NAMPT targeting antitumor agents. Moreover, novel fluorescent probe of NAMPT was identified for the first time. The results highlighted M049–0244 as an antitumor lead compound and a fluorescent probe for imaging NAMPT in living cells.

## Methods

### Human NAMPT protein expression and purification

cDNA sequence of human NAMPT was amplified by PCR from pGex-6p-3-hNAMPT plasmid (kindly gift from Dr. Shui-Qing Ye in University of Missouri) using the following primers: forward, 5′-GGACATATGATGAATCCTGCGGCAGAAGC-3′; and reverse, 5′-AATCTCGA -GGTAATGATGTGCTGCTTCCAGTTC-3′. The PCR products were digested and cloned into pET21a+ vector using NdeI and XhoI restriction enzyme. The resulting vector was introduced into BL21–CondonPlus (DE3)–RIL. The expression of N-terminal His-tagged NAMPT was induced by 0.5 mM isopropyl-β-D-thiogalactopyranoside at an optical density of 0.6–0.8 at 28 °C for 8 h in 2×YT medium containing 100 μg/ml kanamycin and 37 μg/ml chloramphenicol and then purified with nickel–nitrilotriacetic acid resin (QIAGEN). The purity of the protein was verified more than 90% by sodium dodecyl sulfate–polyacrylamide gel electrophoresis (SDS–PAGE) and Coomassie staining.

### High throughput screening (HTS)

HTS was performed using our previously reported method^14^. 0.5 μl stock of each compound (1 mM DMSO stock) was transferred to a 96-well PCR plate for screening. In the primary screening, 5 ng NAMPT in 20 μl reaction buffer [0.4 mM phosphoribosylpyrophosphate (PRPP, Sigma), 2 mM ATP, 0.02% BSA, 2 mM DTT, 12 mM MgCl_2_ and 50 mM Tris-HCl (pH = 7.5)] was added into each well, the plate was incubated at 37 °C for 5 min, then 4.5 μl substrate of NAM was added to initiate the enzyme reaction, resulting in a final concentration of 2% DMSO, 2 μg/ml NAMPT, 0.2 μM NAM and 20 μM compound. After reacting at 37 °C for 15 min, the enzyme reaction was terminated by heating at 95 °C for 1 min and cooling in an ice bath. The product of NMN was detected through the following approach: after adding 10 μl 20% acetophenone in DMSO and 10 μl 2 M KOH into each well, the mixture was vortex-mixed and kept in ice bath for 2 min. Then 45 μl 88% formic acid was added and the mixture was incubated at 37 °C for 10 min. Finally, 85 μl mixtures in each well were transferred into a flat-bottom 96-well black plate (Greiner), and the fluorescence (F) was measured using a Tecan Infinity M200 plate reader (Tecan Group Ltd.) by setting the excitation and emission wavelength to 382 nm and 445 nm respectively. The last row of each 96-well plate includes six wells of background controls and six wells of reference controls. The relative enzyme activity (Activity%) regulated by specific compound was calculated according to equation (1):


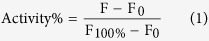


F_0_ was the averaged fluorescence of six background controls, representing zero activity from a simulated enzyme reaction with only NAMPT but no NAM and compound; F_100%_ was the averaged fluorescence of six reference controls, representing 100% activity from intact enzyme reaction without compound perturbation.

Compounds with Activity% less than 40% were considered as inhibitors and subjected to a secondary screen inhibition validation, in which another background control (F_C0_), a simulated enzyme reaction with NAMPT and compound but no NAM, was introduced to eliminate the direct and/or indirect influence from compound. At this stage, the Activity% was calculated according to equation (2):


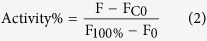


In the screening, the signal-to-noise (S/N) ratio was calculated using the equation: (Mean _signal_ -Mean _background_) / SD _background_^25^. Coefficients of variation (CV) were the ratio of SD to mean. The Z’ factor was determined by equation (3)^26^:


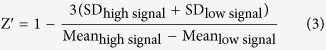


### Determination of IC_50_ for NAMPT inhibitors

To determine the IC_50_ of inhibitors, 0.5 μl compound stocks with various concentrations were added into 96-well plate. The plate was incubated at 37 °C for 5 min after addition of 20 μl reaction buffer containing NAMPT. The enzyme reactions were initiated by 4.5 μl NAM (1.11 μM) following NMN measurement as described above. The IC_50_ values were determined by non-linear fitting of the concentration-dependent curves with the four-parameter IC_50_ logistic equation.

### Cell viability assay and determination of IC_50_ for anti-proliferative effects of NAMPT inhibitors

Cell viability was determined by our previous method^27^ using the Cell Counting Kit-8 (CCK-8, Dojindo, Japan). In human hepatocellular carcinoma cell line HepG2, cells were seeded in 96-well plate and starved for over 12 h with serum-free DMEM at 60~70% confluency, then treated with compounds or vehicle for 48 h. To determine the IC_50_ values of inhibitors, compounds with various concentrations were added into the 96-well plate. 10 μl CCK-8 solution was added to the culture medium and incubated at 37 °C for 1 h. The absorbance at 450 nm (A450) was detected by a plate reader. Each experiment was carried out in triplicate in three replicate wells. The IC_50_ values were determined by non-linear fitting of the concentration-dependent curves with the four-parameter IC_50_ logistic equation.

### NAD measurement

Cellular level of NAD was measured by spectrophotometric enzymatic cycling assay, as described previously^28^,^29^. Briefly, cells were seeded in 96-well plate and starved for over 12 h with serum-free DMEM at 60~70% confluency, following by treatment with compounds or vehicle for 24 h. Cells were lysed with 50 μl of 1M HClO_4_ on ice for 30 min. The lysates were cleared by centrifuging at 4 °C at 18,000 × g for 5 min, and cleared lysates (40 μl) were neutralized by adding 1M K_2_CO_3_ (16 μl) and incubation on ice for 20 min. After centrifuging for 10 min, 10 μl of supernatant were mixed with reaction buffer [50 mM Tris-HCl (pH 7.5), 3% ethanol, 1.66 mM PES (phenazineethosulfate), 0.42 mM MTT (3-(4,5-dimethylthiazolyl-2)-2,5-diphenyltetrazolium bromide), 90 μg/ml ADH] in a total volume of 100 ul, and incubated at 37 °C for 40 min. The absorbance at 570 nm was determined. A blank measurement without ADH was also carried out. Each experiment was carried out in triplicate in three replicate wells

### siRNA transfection

NAMPT knockdown in HepG2 cells was performed by transfection of NAMPT siRNA synthesized by Shanghai GenePharma Co., Ltd (Shanghai, China). The siRNA sequence targeting NAMPT corresponded to coding regions (5′-GCAGAACACAGUACCAUAATT-3′, 5′-UUAUGGUACUGUGUUCUGCTT-3′) of the NAMPT gene. The nonspecific siRNA oligonucleotides (synthesized by Shanghai GenePharma Co., Ltd, Shanghai, China) were used as negative controls. 10 nM of NAMPT siRNA or nonspecific siRNA were transfected into HepG2 cells with Lipofectamine RNAiMax reagent (Invitrogen, CA, USA) according to the manufacturer’s instructions. After 24 or 48 hours, transfected cells were subjected to subsequent analysis.

### Quantitative Real-Time PCR

HepG2 cells were seeded in 12-well plates at cell density of 1 × 10^6^ cells/ml and transfected 10 nM of NAMPT siRNA or nonspecific siRNA. After 24 hours, cells was harvested and RNA was extracted using trazole reagent (Invitrogen). Later mRNA was reverse transcribed using PrimeScriptTM RT Master Mix (Takara). Relative amounts of specific cDNAs were quantified by using their specific primers with the SYBR green PCR master mix reagent (Applied Biosystems). The primers used in this study were as follows: NAMPT (5′ AATGTTCTCTTCACGGTGGAAAA 3′ and 5′ACTGTGATTGGATACCAGGACT 3′); ACTIN(TGACAGGATGCAGAAGGAGA and CGCTCAGGAGGAGCAATG)

### Immunoblotting

Tissues were lysated and immunoblotting as described^27^. Blots were incubated with primary antibodies specific for NAMPT (Santa Cruz, sc-67020, used at 1:500) and IRDye800CW-conjugated secondary antibody. The image was captured by the Odyssey infrared imaging system (Li-Cor Bioscience, Lincoln, NE).

### Immunofluorescence

Immunofluorescence were performed as described^27^. Cultured cells were fixed in 4% paraformaldehyde, blocked by 8% normal goat serum, and incubated in specific primary antibody NAMPT (1:200, Santa cruze) .after being washed 3 times with phosphate-buffered saline (PBS), cells were incubated with Alexa 549-conjugated secondary antibody. Images were obtained by fluorescence microscope (IX-71; Olympus, Tokyo, Japan) with a digital camera (Olympus).

### Primary hepatocytes culture

Hepatocytes were isolated from mouse liver by the two-step collagenase perfusion method^30^,^31^. Briefly, Hank’s buffered salt solution followed by collagenase digestion medium was perfused through a cannula inserted from superior vena cava. For imaging experiment, 1 × 10^5^ cells were plated in a 35-mm glass bottom dishes or 5 × 10^4^ were plated in 48-well plate for cell viability assay in DMEM supplemented with 10% FBS and antibiotics, and kept under an atmosphere of 95% air and 5% CO_2_ at 37 °C.

### Imaging of compounds in living cells

For imaging experiment, HepG2 cells or primary hepatocytes were seeded into 35-mm glass bottom dishes (*In Vitro* Scientific). The following day, compounds were added to the medium at a final concentration of 3 μM. After 30 minutes, cells were rinsed twice with PBS (pH 7.5), then incubated in fresh culture medium and imaged on a fluorescence microscope (IX-71, Olympus) with a digital camera (Olympus). After 4 hours, these cells were imaged once again. Digital images were recorded and analyzed using ImageJ.

## Additional Information

**How to cite this article**: Wang, X. *et al.* Discovery of Novel Inhibitors and Fluorescent Probe Targeting NAMPT. *Sci. Rep.* **5**, 12657; doi: 10.1038/srep12657 (2015).

## Supplementary Material

Supplementary Information

Supplemental dataset S1

Supplemental dataset S2

Supplemental dataset S3

## Figures and Tables

**Table 1:**
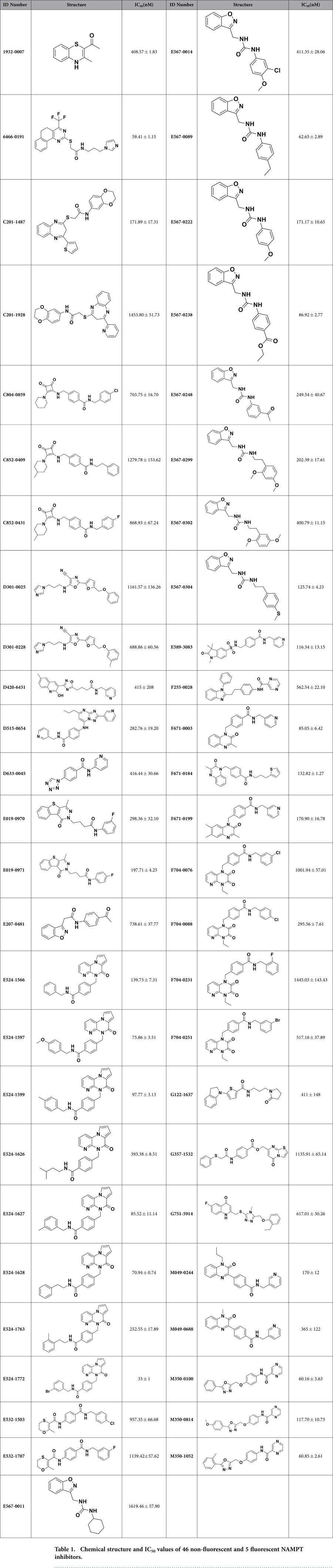
Chemical structure and IC_50_ values of 46 non-fluorescent and 5 fluorescent NAMPT inhibitors.

## References

[b1] Garten A., Petzold S., Korner A., Imai S. & Kiess W. Nampt: linking NAD biology, metabolism and cancer. Trends Endocrinol. Metab. 20, 130–138 (2009). 19109034 10.1016/j.tem.2008.10.004 PMC2738422

[b2] Dahl T. B., Holm S., Aukrust P. & Halvorsen B. Visfatin/NAMPT: a multifaceted molecule with diverse roles in physiology and pathophysiology. Annu. Rev. Nutr. 32, 229–243 (2012). 22462624 10.1146/annurev-nutr-071811-150746

[b3] Wang P., Vanhoutte P. M. & Miao C. Y. Visfatin and cardio-cerebro-vascular disease. J. Cardiovasc. Pharmacol. 59, 1–9 (2012). 21266913 10.1097/FJC.0b013e31820eb8f6

[b4] Buldak R. J. *et al.* Visfatin affects redox adaptative responses and proliferation in Me45 human malignant melanoma cells: an *in vitro* study. Oncol. Rep. 29, 771–778 (2013). 23232726 10.3892/or.2012.2175

[b5] Miao C. Y. Introduction: Adipokines and cardiovascular disease. Clin. Exp. Pharmacol. Physiol. 38, 860–863 (2011). 21883383 10.1111/j.1440-1681.2011.05598.x

[b6] Galli U. *et al.* Medicinal chemistry of nicotinamide phosphoribosyltransferase (NAMPT) inhibitors. J. Med. Chem. 56, 6279–6296 (2013). 23679915 10.1021/jm4001049

[b7] Ravaud A. *et al.* Phase I study and pharmacokinetic of CHS-828, a guanidino-containing compound, administered orally as a single dose every 3 weeks in solid tumours: an ECSG/EORTC study. Eur. J. Cancer 41, 702–707 (2005). 15763645 10.1016/j.ejca.2004.12.023

[b8] Holen K., Saltz L. B., Hollywood E., Burk K. & Hanauske A. R. The pharmacokinetics, toxicities, and biologic effects of FK866, a nicotinamide adenine dinucleotide biosynthesis inhibitor. Invest New Drugs 26, 45–51 (2008). 17924057 10.1007/s10637-007-9083-2

[b9] von Heideman A., Berglund A., Larsson R. & Nygren P. Safety and efficacy of NAD depleting cancer drugs: results of a phase I clinical trial of CHS 828 and overview of published data. Cancer Chemother. Pharmacol. 65, 1165–1172 (2010). 19789873 10.1007/s00280-009-1125-3

[b10] Matheny C. J. *et al.* Next-generation NAMPT inhibitors identified by sequential high-throughput phenotypic chemical and functional genomic screens. Chem. Biol. 20, 1352–1363 (2013). 24183972 10.1016/j.chembiol.2013.09.014 PMC3881547

[b11] Zheng X. *et al.* Structure-based identification of ureas as novel nicotinamide phosphoribosyltransferase (Nampt) inhibitors. J. Med. Chem. 56, 4921–4937 (2013). 23617784 10.1021/jm400186h

[b12] Christensen M. K. *et al.* Nicotinamide phosphoribosyltransferase inhibitors, design, preparation, and structure-activity relationship. J. Med. Chem. 56, 9071–9088 (2013). 24164086 10.1021/jm4009949

[b13] Giannetti A. M. *et al.* Fragment-based identification of amides derived from trans-2-(pyridin-3-yl)cyclopropanecarboxylic acid as potent inhibitors of human nicotinamide phosphoribosyltransferase (NAMPT). J. Med. Chem. 57, 770–792 (2014). 24405419 10.1021/jm4015108

[b14] Zhang R. Y. *et al.* A fluorometric assay for high-throughput screening targeting nicotinamide phosphoribosyltransferase. Anal. Biochem. 412, 18–25 (2011). 21211508 10.1016/j.ab.2010.12.035

[b15] Xu T. Y. *et al.* Discovery and characterization of novel small-molecule inhibitors targeting nicotinamide phosphoribosyltransferase. Sci. Rep. 5, 10043 (2015). 26040985 10.1038/srep10043 PMC4603696

[b16] De La Fuente J. R., Canete A., Zanocco A. L., Saitz C. & Jullian C. Formal hydride transfer mechanism for photoreduction of 3-phenylquinoxalin-2-ones by amines. Association Of 3-phenylquinoxalin-2-one with aliphatic amines. J. Org. Chem. 65, 7949–7958 (2000). 11073603 10.1021/jo000992r

[b17] Wang P. *et al.* Intracellular NAMPT-NAD+-SIRT1 cascade improves post-ischaemic vascular repair by modulating Notch signalling in endothelial progenitors. Cardiovasc. Res. 104, 477–488 (2014). 25341895 10.1093/cvr/cvu220

[b18] Martinez Molina D. *et al.* Monitoring drug target engagement in cells and tissues using the cellular thermal shift assay. Science 341, 84–87 (2013). 23828940 10.1126/science.1233606

[b19] Schuster S. *et al.* FK866-induced NAMPT inhibition activates AMPK and downregulates mTOR signaling in hepatocarcinoma cells. Biochem. Biophys. Res. Commun. 458, 334–340 (2015). 25656579 10.1016/j.bbrc.2015.01.111

[b20] Hasmann M. & Schemainda I. FK866, a highly specific noncompetitive inhibitor of nicotinamide phosphoribosyltransferase, represents a novel mechanism for induction of tumor cell apoptosis. Cancer Res. 63, 7436–7442 (2003). 14612543

[b21] Sampath D., Zabka T. S., Misner D. L., O’Brien T. & Dragovich P. S. Inhibition of nicotinamide phosphoribosyltransferase (NAMPT) as a therapeutic strategy in cancer. Pharmacol. Ther. 151, 16–31 (2015). 25709099 10.1016/j.pharmthera.2015.02.004

[b22] Morris-Blanco K. C., Cohan C. H., Neumann J. T., Sick T. J. & Perez-Pinzon M. A. Protein kinase C epsilon regulates mitochondrial pools of Nampt and NAD following resveratrol and ischemic preconditioning in the rat cortex. J. Cereb. Blood Flow Metab. 34, 1024–1032 (2014). 24667915 10.1038/jcbfm.2014.51 PMC4050248

[b23] Yang H. *et al.* Nutrient-sensitive mitochondrial NAD+ levels dictate cell survival. Cell 130, 1095–1107 (2007). 17889652 10.1016/j.cell.2007.07.035 PMC3366687

[b24] Pittelli M. *et al.* Inhibition of nicotinamide phosphoribosyltransferase: cellular bioenergetics reveals a mitochondrial insensitive NAD pool. J. Biol. Chem. 285, 34106–34114 (2010). 20724478 10.1074/jbc.M110.136739 PMC2962509

[b25] Rowlands M. G. *et al.* High-throughput screening assay for inhibitors of heat-shock protein 90 ATPase activity. Anal. Biochem. 327, 176–183 (2004). 15051534 10.1016/j.ab.2003.10.038

[b26] Zhang J. H., Chung T. D. & Oldenburg K. R. A Simple Statistical Parameter for Use in Evaluation and Validation of High Throughput Screening Assays. J. Biomol. Screen 4, 67–73 (1999). 10838414 10.1177/108705719900400206

[b27] Wang P. *et al.* Perivascular adipose tissue-derived visfatin is a vascular smooth muscle cell growth factor: role of nicotinamide mononucleotide. Cardiovasc. Res. 81, 370–380 (2009). 18952695 10.1093/cvr/cvn288

[b28] Matsumura H. & Miyachi S. Cycling Assay for Nicotinamide Adenine Dinucleotides. Methods In Enzymology 69, 465–470 (1980).

[b29] Wang P. *et al.* Loss of AMP-activated protein kinase-alpha2 impairs the insulin-sensitizing effect of calorie restriction in skeletal muscle. Diabetes 61, 1051–1061 (2012). 22396207 10.2337/db11-1180 PMC3331748

[b30] Klaunig J. E. *et al.* Mouse liver cell culture. I. Hepatocyte isolation. In Vitro 17, 913–925 (1981). 6273298 10.1007/BF02618288

[b31] Klaunig J. E., Goldblatt P. J., Hinton D. E., Lipsky M. M. & Trump B. F. Mouse liver cell culture. II. Primary culture. In Vitro 17, 926–934 (1981). 7309042 10.1007/BF02618289

