# Supplementary material for: Discovery of Novel Inhibitors and Fluorescent Probe Targeting NAMPT
Source: Sci Rep. 2015 Jul 31;5:12657. doi: 10.1038/srep12657 (PMC4521150; doi:10.1038/srep12657)
Supplement: Supplementary Information [file srep12657-s1.doc]

**Supplemental Information**

**Discovery of Novel Inhibitors and Fluorescent Probe Targeting NAMPT**

Xia Wang,^†,#^ Tian-Ying Xu,^†,#^ Xin-Zhu Liu,^†,#^ Sai-Long Zhang,^†^ Pei Wang,^†^ Zhi-Yong Li,^†^ Yun-Feng Guan,^†^ Shu-Na Wang,^†^ Guo-Qiang Dong,^‡^ Shu Zhuo,^§^ Ying-Ying Le,^§^ Chun-Quan Sheng,*^,‡^ and Chao-Yu Miao*^,†^

^†^Department of Pharmacology, and ^‡^Department of Medicinal Chemistry, Second Military Medical University, Shanghai, China; ^§^Key Laboratory of Nutrition and Metabolism, Institute for Nutritional Sciences, Shanghai Institutes for Biological Sciences, Chinese Academy of Sciences, Shanghai, China

The first three authors contributed equally to this work.

*Corresponding author:

E-mail: cymiao@smmu.edu.cn or shengcq@hotmail.com

**Supplemental Data**

**Figure S1. Western blot analysis of the NAMPT protein in HepG2 cells.**


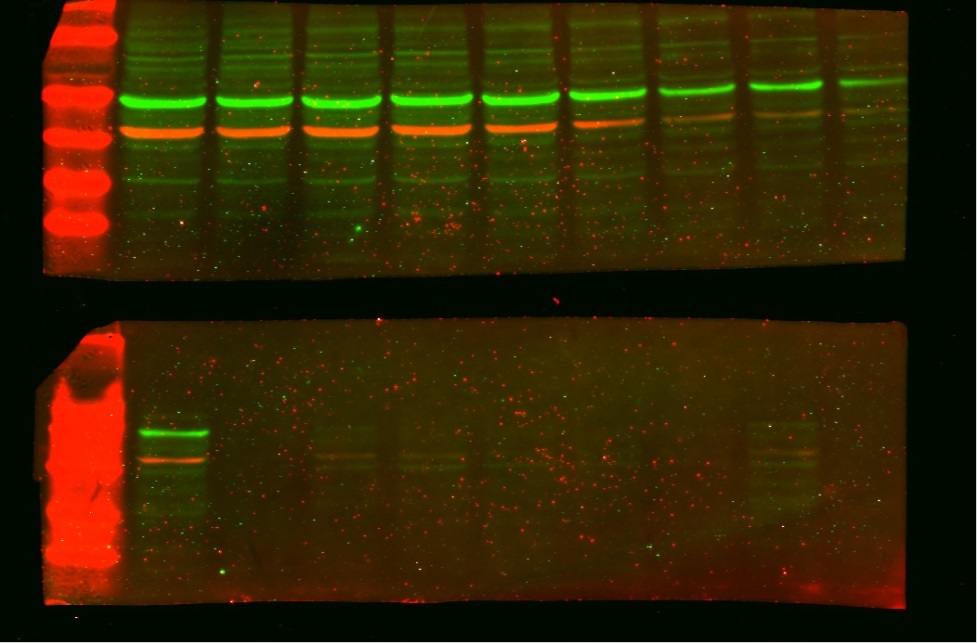


**Marker**

**NAMPT**

**55KDa**

**43KDa**

**β-actin**

**Figure S2. Effect of F671-0003 and M049-0244 on Huh-7 cell viability.**

**P*<0.05, ***P*<0.01 vs serum free medium control. Data are shown as mean±SEM.


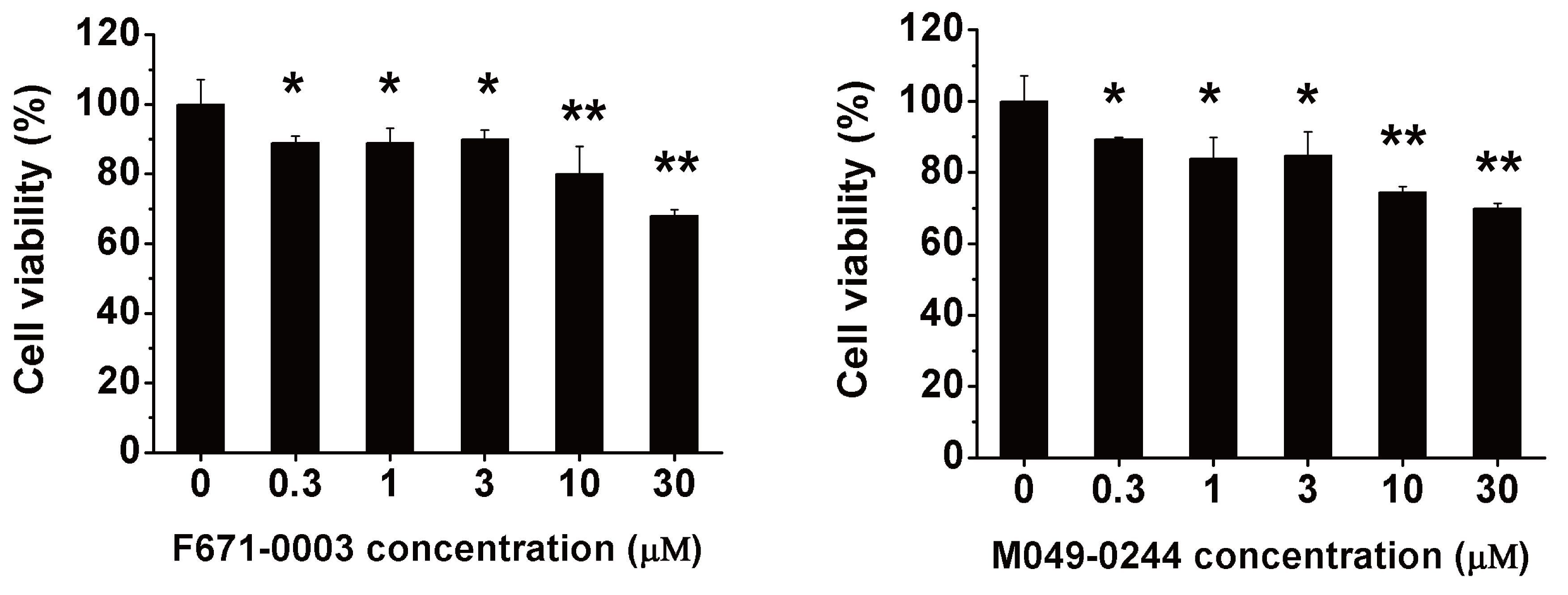


**Figure S3. Representative images of fluorescent molecule M049-0244 in HepG2 cells treated for different periods of time at 3 µM or treated for same time of 30 minutes at various concentrations.**


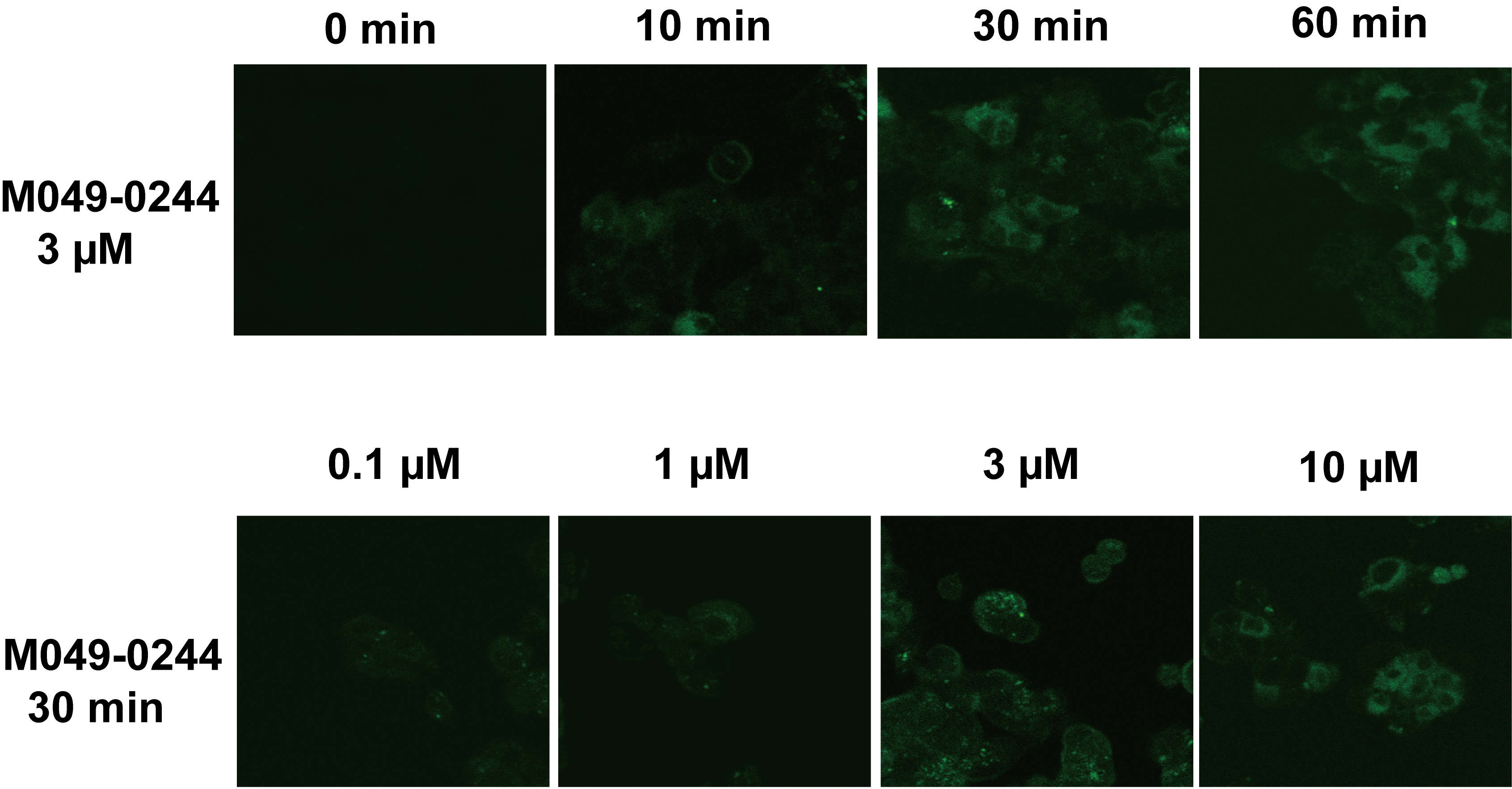


**Figure S4. Superimposition of the binding conformation of F671-0003 and M049-0244 with FK866 and CHS828 in the active site of NAMPT.**


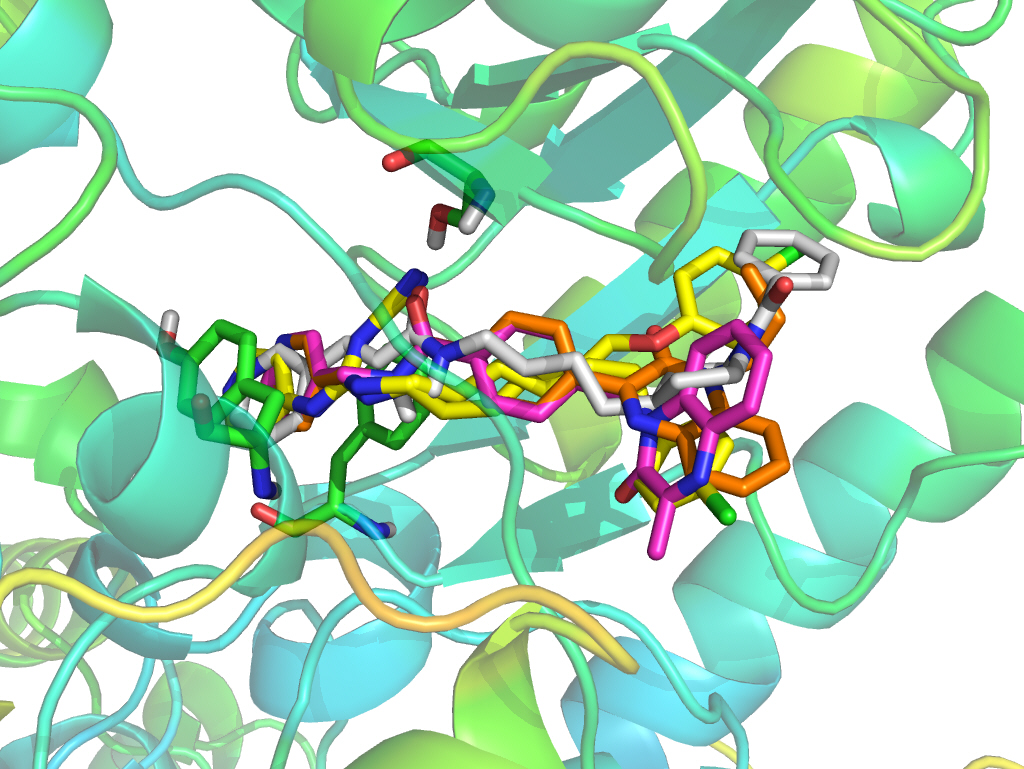


**FK866 (White)**

**CHS828 (Yellow)**

**F671-0003 (Purple)**

**M049-0244 (Orange)**

**Table S1. Similarity index of F671-0003 and M049-0244 with the reported NAMPT inhibitors.**

| **Compound** | **Compound** | **Similarity index** |
| --- | --- | --- |
| **F671-0003** | **FK866** | 0.18 |
| **F671-0003** | **CHS828** | 0.12 |
| **F671-0003** | **GNE-617** | 0.18 |
| **F671-0003** | **STF118804** | 0.34 |
| **M049-0244** | **FK866** | 0.20 |
| **M049-0244** | **CHS828** | 0.14 |
| **M049-0244** | **GNE-617** | 0.17 |
| **M049-0244** | **STF118804** | 0.33 |

**Table S2**. The calculated physicochemical properties of NAMPT inhibitors

| **Name** | **MW** | **ALogP** | **Num_H_acceptors** | **Num_H_Donors** |
| --- | --- | --- | --- | --- |
| **M049-0244** | 398.46 | 3.22 | 4 | 1 |
| **F671-0003** | 384.43 | 2.26 | 6 | 2 |
| **FK866** | 391.51 | 3.27 | 3 | 1 |
| **CHS828** | 371.86 | 3.84 | 6 | 2 |

MW, molecular weight.
